# Metabolic Myopathies: “Human Knockout” Models and Translational Medicine

**DOI:** 10.3389/fphys.2020.00350

**Published:** 2020-04-30

**Authors:** Bruno Grassi, Simone Porcelli, Mauro Marzorati

**Affiliations:** ^1^Department of Medicine, University of Udine, Udine, Italy; ^2^Institute of Biomedical Technologies, National Research Council, Segrate, Italy; ^3^Department of Molecular Medicine, University of Pavia, Pavia, Italy

**Keywords:** metabolic myopathies, mitochondrial myopathies, McArdle disease, functional evaluation, skeletal muscle oxidative metabolism

Metabolic myopathies are rare diseases characterized by derangements of glycogen or lipid metabolism or mitochondrial function, caused by genetic mutations leading to defects of the main pathways of energy provision in skeletal muscle fibers.

The patients can be considered a sort of “human knockout” model, and present unique opportunities to investigate fundamental physiological mechanisms. One of the first authors to exploit this possibility was James Hagberg in the early 1980's, in a classic paper (Hagberg et al., 1982) carried out on patients with McArdle disease (McA). In McA patients the absence of myophosphorylase activity substantially impairs the flux of substrates along the glycolytic pathway (Lewis and Haller, 1986). At the end of an incremental exercise these patients typically reach exhaustion without any increase in blood lactic acid levels vs. those determined at rest (Hagberg et al., 1982), and no metabolic acidosis ensues during exercise. Hagberg et al. (1982) demonstrated that the “ventilatory threshold” (pulmonary ventilation [V°E] and CO_2_ output [V°CO_2_] “disproportionate increases” vs. that of O_2_ uptake [V°O_2_], occurring at about 50–70% of peak work rate) (Beaver et al., 1986) was not different in McA patients vs. controls, providing evidence against a causative role for blood lactic acid in the V°E and V°CO_2_ responses.

Mechanisms of metabolic regulation often present a redundancy, and within a complex system several factors may concur in determining a response. Thus, in strict terms the experimental “elimination” of one factor, and the observation of a normal response, does not mean that the factor, in physiological conditions, does not have a regulatory role. Nonetheless, in the example mentioned above [identification of the factor[s] responsible for the ventilatory threshold], McA patients represent indeed an ideal experimental model. Let's summarize the *scenario*. Experimental question: are the disproportionate increases in V°E and V°CO_2_, vs. that of V°O_2_, observed during an incremental exercise, attributable to an increased elimination of CO_2_ generated in tissues and blood as a consequence of H^+^ buffering by bicarbonate? The experimental approach was straightforward: what happens in McA patients, in whom (without the need of any experimental intervention) no H^+^ accumulation in muscles and blood occurs during exercise? The experimental data were clear: no differences for the V°E or V°CO_2_ vs. V°O_2_ responses in the McA patients vs. the controls. Take home message: H^+^ accumulation in blood is not necessary for the V°E or V°CO_2_ vs. V°O_2_ disproportionate increases observed at 50-70% of peak work rate during an incremental exercise (Hagberg et al., 1982).

In McA patients the reduced flux of substrates along the glycolytic pathway limits the supply of substrates to the tricarboxylic acid cycle, thereby impairing oxidative metabolism, as also suggested by the slow V°O_2_ kinetics during the transition from rest to exercise (Grassi et al., 2009) and by the slow phosphocreatine recovery kinetics during the recovery from exercise (Siciliano et al., 1995). A similar impairment, although by a different and more “downstream” cause (mutations leading to impairment[s] of enzyme[s] of the mitochondrial respiratory chain) is described in patients affected by another type of metabolic myopathy, the heterogeneous series of diseases termed “mitochondrial myopathies” (MM). Also in this respect McA and MM patients may function as human knockout models, allowing to elucidate basic physiological mechanisms.

An excellent example is represented by a set of compensatory responses occurring in the framework of a physiological adaptation, described over the years, thanks to the elegant work of different groups. In MM and McA patients the impaired oxidative metabolism [considered as whole body “peak” pulmonary V°O_2_ (Linderholm et al., 1969; Lewis and Haller, 1986; Taivassalo et al., 2003; Grassi et al., 2007), or as muscle peak V°O_2_ (Taivassalo et al., 2002)] is almost exclusively attributable to an impaired peak capacity of fractional O_2_ extraction, i.e., to an impairment of the second term of the right-hand portion of the equation describing the Fick principle of conservation of mass (systemic arterial—mixed venous O_2_ concentration difference, C(a-$\bar{v}$)O_2_; or “local” arterial—venous O_2_ concentration difference, C(a-v)O_2_). After putting fractional O_2_ extraction in the equation expressing the Fick principle we obtain, at the whole body level:

(1) $$\begin{array}{r} \text{Pulmonary }\overset{∙}{\text{V}}O_{2}\;=\text{ cardiac output }\times \;C\left(a-\bar{v}\right)O_{2} \end{array}$$

On the other hand, if we consider the Fick principle of conservation of mass across a specific skeletal muscle group we obtain:

(2) $$\begin{array}{r} \text{Muscle }\overset{∙}{\text{V}}O_{2}=\text{muscle blood flow }\times \;C\left(a-v\right)O_{2} \end{array}$$

Several groups have documented decreased submaximal and peak C(a-$\bar{v}$)O_2_ in MM (Linderholm et al., 1969; Taivassalo et al., 2003) and McA (Lewis and Haller, 1986) patients, mainly by measuring cardiac output and V°O_2_ during an incremental test. Other groups demonstrated a decreased submaximal and peak C(a-v)O_2_ across an exercising limb (Taivassalo et al., 2002), by invasive measurements of CaO_2_ and CvO_2_, or indirectly by determining submaximal and peak fractional O_2_ extraction in muscle by near-infrared spectroscopy (NIRS, see the review by Grassi and Quaresima, 2016) (Grassi et al., 2007).

These authors also described a highly significant and negative linear correlation between peak V°O_2_ and the NIRS-determined peak fractional O_2_ extraction (Grassi et al., 2007). Although a correlation does not imply cause-effect, within the patho-physiological context the data strongly suggest that in MM and McA patients the impaired peak fractional O_2_ extraction is presumably the cause of the impaired peak V°O_2_ (Grassi et al., 2007).

Oxidative metabolism is essential to sustain activities of exeryday life. Thus, it is reasonable to expect some compensatory mechanisms entailed by MM and McA patients in order to restore pulmonary or muscle V°O_2_ values, or at least to attenuate the V°O_2_ impairments attributable to the disease. How to accomplish this? The answer lies again in the equations expressing the Fick principle of conservation of mass mentioned above (Equations 1, 2): if the second term of the right-hand portion of the equations (fractional O_2_ extraction) is impaired, the system tries to compensate for this impairment by increasing the first term of the right-hand portion of the equations (O_2_ delivery), that is cardiac output or muscle blood flow.

This compensatory mechanism has been repeatedly demonstrated in MM and in McA patients, starting from the description, in the pioneering work by Linderholm et al. (1969), of the “exaggerated” or “hyperkinetic” cardiovascular response to exercise. Higher O_2_ delivery values vs. controls, for the same work rate, were subsequently described both in MM (see e.g., Taivassalo et al., 2002, 2003) and in McA patients (see e.g., Lewis and Haller, 1986). Grassi et al. (2007) considered the heart rate (HR) response a reasonable “proxy” of the cardiac output response, and described steeper HR vs. work rate relationships in McA and MM patients vs. controls. Interestingly, the slopes of the HR vs. work rate relationships were linearly related to the impairment of fractional O_2_ extraction, as estimated by NIRS (Grassi et al., 2007). In other words, in the presence of a more severe impairment of oxidative metabolism, a more pronounced cardiovascular response was described; the signals involved in this “metaboreflex” are presumably different in MM and in McA patients. Enhanced systemic norepinephrine vasoconstriction and enhanced functional sympatholysis in working muscles have been described in MM patients (see e.g., Jeppesen and Vissing, 2019); in these patients a preminent role in peripheral vasodilation would be played by the ATP released by red blood cells (Jeppesen and Vissing, 2019).

The compensatory mechanism of enhanced O_2_ delivery would manifest itself also at a morphological level. Taivassalo et al. (2012) have indeed demonstrated that in MM patients the iperkinetic cardiovascular response was associated with increased capillary and vascular angiogenic growth factor levels in skeletal muscles, the increased capillarity being more pronounced around the fibers with the most pronounced oxidative impairment. In other words, besides inducing an enhanced cardiovascular response the impaired skeletal muscle oxidative metabolism would also promote angiogenesis. Another beautiful example of physiological mechanisms uncovered by the study of the human knockout models.

Apart from the algebraic approach (see above), what would the physiological rationale be of increasing O_2_ delivery to muscle fibers, which have problems in utilizing the O_2_ they receive from the cardiovascular system? Porcelli et al. (2019) recently proposed an answer to this question by applying to MM patients the “Wagner's approach” (Wagner, 1996) evaluating perfusive and diffusive limitations in the O_2_ pathway. In Figure 3 of that paper (Porcelli et al., 2019), peak O_2_ delivery (cardiac output × CaO_2_) in MM patients was considered to be normal (see Linderholm et al., 1969; Taivassalo et al., 2003). In the model peripheral O_2_ diffusion is dictated by the Fick law of diffusion:

(3) $$\begin{array}{r} \overset{∙}{\text{V}}O_{2}\;=\text{ D}m{\text{O}}_{2}\;\times \;\left(\text{P}m\text{v}O_{2}-PiO_{2}\right) \end{array}$$

in which D*m*O_2_ represents peripheral O_2_ diffusive capacity, P*mv*O_2_ microvascular partial pressure of O_2_ and P*i*O_2_ intracellular PO_2_. In MM and McA patients V°O_2_ at peak exercise is lower than normal because peripheral O_2_ diffusion is impaired (see Figure 3 in Porcelli et al., 2019). This impairment occurs because P*i*O2 is presumably higher than normal (impaired intracellular oxidative metabolism), thereby decreasing the PO_2_ gradient from the microvascular to the intracellular compartment. In this *scenario*, how can the partial pressures gradient be increased, thereby enhancing peripheral O_2_ diffusion? The answer: an enhanced O_2_ delivery would increase P*mv*O_2_, thereby increasing the driving pressure for the peripheral diffusion of the gas.

Whereas, at submaximal work rates the increased O_2_ delivery can fully compensate for the impaired fractional O_2_ extraction (V°O_2_ being therefore substantially normal, for the same submaximal work rate, between patients and controls), the compensation may not be complete in “maximal” or “peak” conditions, that is when the patient reaches exhaustion. This translates into a lower peak V°O_2_, with the associated impaired exercise tolerance. Peak V°O_2_ values in MM and McA patients, although presenting a substantial variability among patients, are typically around 10–20 ml kg^−1^ min^−1^ (see e.g., Lewis and Haller, 1986; Taivassalo et al., 2003; Grassi et al., 2007; Porcelli et al., 2016, 2019), i.e., in a range compatible with patients belonging to NYHA class II-IV heart failure.

In conclusion, we hope we have convinced the reader that the human knockout models of MM and McA patients are ideally suited to be evaluated within a translational approach, which brings basic science methods and tools “to the bed of the patient.” This can be accomplished by utilizing non-invasive experimental approaches developed over the years in the field of exercise physiology. In these patients the aims are to address specific issues related to basic pathophysiological mechanisms, to identify and quantify the impairment of skeletal muscle oxidative metabolism and the factors limiting exercise tolerance, and ultimately the patients' quality of life. The non-invasiveness of the adopted methods facilitates serial measurements, allowing clinical course of the diseases to be examined, as well as the efficacy of rehabilitation or therapeutical interventions.

Physiology and physiological research remain the essential link between genes, molecules and clinical care (Joyner, 2011; Wagner and Paterson, 2011). The –omics world may identify concepts and mechanisms, but only physiology can give a meaning to these concepts and mechanisms within the general picture of a human body, healthy or ill (Grassi et al., 2019).

## Author Contributions

BG, SP, and MM participated to the studies mentioned in the Opinion. BG wrote the first draft of the present manuscript. SP and MM participated in the discussion and in the editing of the manuscript.

## Conflict of Interest

The authors declare that the research was conducted in the absence of any commercial or financial relationships that could be construed as a potential conflict of interest.
